# Parotid-sparing intensity modulated versus conventional radiotherapy in head and neck cancer (PARSPORT): a phase 3 multicentre randomised controlled trial

**DOI:** 10.1016/S1470-2045(10)70290-4

**Published:** 2011-02

**Authors:** Christopher M Nutting, James P Morden, Kevin J Harrington, Teresa Guerrero Urbano, Shreerang A Bhide, Catharine Clark, Elizabeth A Miles, Aisha B Miah, Kate Newbold, MaryAnne Tanay, Fawzi Adab, Sarah J Jefferies, Christopher Scrase, Beng K Yap, Roger P A'Hern, Mark A Sydenham, Marie Emson, Emma Hall, on behalf of the PARSPORT trial management group

**Affiliations:** a Head and Neck Unit, Royal Marsden Hospitals NHS Foundation Trust, London, UK; b Clinical Trials and Statistics Unit, The Institute of Cancer Research, Sutton, Surrey, UK; c Department of Oncology, Guy's and St Thomas' NHS Foundation Trust, London, UK; d Department of Medical Physics, Royal Surrey County Hospital NHS Foundation Trust, Guildford, UK; e National Radiotherapy Trials QA Group, Mount Vernon Hospital, Northwood, UK; f Cancer Centre, University Hospital of North Staffordshire NHS Trust, Stoke on Trent, UK; g Oncology Centre, Addenbrooke's Hospital NHS Foundation Trust, Cambridge, UK; h Department of Clinical Oncology, The Ipswich Hospital NHS Trust, Ipswich, UK; i Department of Radiotherapy, The Christie NHS Foundation Trust, Manchester, UK

## Abstract

**Background:**

Xerostomia is the most common late side-effect of radiotherapy to the head and neck. Compared with conventional radiotherapy, intensity-modulated radiotherapy (IMRT) can reduce irradiation of the parotid glands. We assessed the hypothesis that parotid-sparing IMRT reduces the incidence of severe xerostomia.

**Methods:**

We undertook a randomised controlled trial between Jan 21, 2003, and Dec 7, 2007, that compared conventional radiotherapy (control) with parotid-sparing IMRT. We randomly assigned patients with histologically confirmed pharyngeal squamous-cell carcinoma (T1–4, N0–3, M0) at six UK radiotherapy centres between the two radiotherapy techniques (1:1 ratio). A dose of 60 or 65 Gy was prescribed in 30 daily fractions given Monday to Friday. Treatment was not masked. Randomisation was by computer-generated permuted blocks and was stratified by centre and tumour site. Our primary endpoint was the proportion of patients with grade 2 or worse xerostomia at 12 months, as assessed by the Late Effects of Normal Tissue (LENT SOMA) scale. Analyses were done on an intention-to-treat basis, with all patients who had assessments included. Long-term follow-up of patients is ongoing. This study is registered with the International Standard Randomised Controlled Trial register, number ISRCTN48243537.

**Findings:**

47 patients were assigned to each treatment arm. Median follow-up was 44·0 months (IQR 30·0–59·7). Six patients from each group died before 12 months and seven patients from the conventional radiotherapy and two from the IMRT group were not assessed at 12 months. At 12 months xerostomia side-effects were reported in 73 of 82 alive patients; grade 2 or worse xerostomia at 12 months was significantly lower in the IMRT group than in the conventional radiotherapy group (25 [74%; 95% CI 56–87] of 34 patients given conventional radiotherapy *vs* 15 [38%; 23–55] of 39 given IMRT, p=0·0027). The only recorded acute adverse event of grade 2 or worse that differed significantly between the treatment groups was fatigue, which was more prevalent in the IMRT group (18 [41%; 99% CI 23–61] of 44 patients given conventional radiotherapy *vs* 35 [74%; 55–89] of 47 given IMRT, p=0·0015). At 24 months, grade 2 or worse xerostomia was significantly less common with IMRT than with conventional radiotherapy (20 [83%; 95% CI 63–95] of 24 patients given conventional radiotherapy *vs* nine [29%; 14–48] of 31 given IMRT; p<0·0001). At 12 and 24 months, significant benefits were seen in recovery of saliva secretion with IMRT compared with conventional radiotherapy, as were clinically significant improvements in dry-mouth-specific and global quality of life scores. At 24 months, no significant differences were seen between randomised groups in non-xerostomia late toxicities, locoregional control, or overall survival.

**Interpretation:**

Sparing the parotid glands with IMRT significantly reduces the incidence of xerostomia and leads to recovery of saliva secretion and improvements in associated quality of life, and thus strongly supports a role for IMRT in squamous-cell carcinoma of the head and neck.

**Funding:**

Cancer Research UK (CRUK/03/005).

## Introduction

Radiotherapy is the main non-surgical treatment for squamous-cell carcinoma of the head and neck (HNSCC).^1^ High rates of local tumour control can be achieved with 5-year survival greater than 80% for stage 1 and 2 and 60–70% for stage 3 and 4 tumours;^2^ however, long-term late sequelae of radiotherapy are highly prevalent and have severe adverse effects on quality of life (QoL).^3^, ^4^ Radiation-induced xerostomia is the most commonly reported late side-effect of radiotherapy to the head and neck. Lack of saliva affects speech and swallowing and can accelerate dental caries.^5^

Intensity-modulated radiotherapy (IMRT) is a conformal radiotherapy technique that can spare the major salivary glands. Small phase 2 studies have shown that a reduction in radiation to the parotid glands (to 24–26 Gy) through parotid-sparing IMRT aids recovery of saliva flow.^6^, ^7^, ^8^ We report results of the first multicentre randomised controlled trial to assess parotid-sparing IMRT in patients with HNSCC.

## Methods

### Participants

We undertook a phase 3 randomised controlled trial at six UK radiotherapy centres (recruitment between Jan 21, 2003, and Dec 7, 2007). Eligible patients had histologically confirmed HNSCC that arose from the oropharynx or hypopharynx and were to be treated by radiotherapy either primarily or postoperatively without concomitant chemotherapy. These patients were at high risk of radiation-induced xerostomia—ie, if they were treated with conventional radiotherapy the estimated mean dose to both parotid glands would be greater than 24 Gy. Patients had WHO performance status 0 or 1 and any stage of disease except M1. Patients were required to attend regular follow-up, undergo salivary flow measurements, and complete self-assessed QoL questionnaires.

Exclusion criteria included previous head or neck radiotherapy; previous malignancy except non-melanoma skin cancer; pre-existing salivary gland disease; tumour involvement of the parotid glands; or previous or concurrent illness that would compromise completion of treatment or follow-up. Prophylactic amifostine or pilocarpine was not permitted. Patients who had received neoadjuvant chemotherapy were eligible.

All patients provided written informed consent. PARSPORT (CRUK/03/005) was approved by the national South-West Multicentre Research Ethics Committee (MREC 03/6/79) and the local ethics committees of all participating centres. Our trial was sponsored by the Royal Marsden NHS Foundation Trust and undertaken in accordance with the principles of Good Clinical Practice.

### Randomisation and masking

Patients were randomly assigned in a 1:1 ratio to parotid-sparing IMRT or conventional radiotherapy (control). Independent randomisation was via telephone to the Clinical Trials and Statistics Unit at the Institute of Cancer Research (ICR-CTSU). Computer-generated random permuted blocks were used; stratification was by treatment centre and tumour site. Treatment allocation was not masked; however, the patient was not informed of the treatment until they had completed the baseline QoL questionnaires.

### Procedures

Staging investigations included examination under anaesthetic, tumour biopsy, diagnostic CT or MRI of head and neck, chest radiograph, full blood count, and biochemistry. In postoperative patients, histology reports that documented the extent of surgical resection were required.

The protocol for target volume definition and treatment planning has been previously described.^9^ All patients underwent CT-planned radiotherapy with either three-dimensional conformal radiotherapy with parallel opposed lateral fields (conventional radiotherapy) or parotid-sparing IMRT. The conventional radiotherapy regimen was the national standard of care in the UK and most other countries at the time our trial was designed. In both treatment groups, the primary tumour and involved lymph nodes were treated with 65 Gy in 30 daily fractions given Monday to Friday. 60 Gy in 30 fractions was delivered to postoperative patients unless there was macroscopic residual disease in which case 65 Gy in 30 fractions was given. Nodal groups at risk of harbouring occult metastatic disease received a biologically equivalent dose of either 50 Gy in 25 daily fractions (conventional radiotherapy) or 54 Gy in 30 fractions (IMRT). For IMRT patients a planning constraint of less than 24 Gy to the whole contralateral parotid gland was used.^9^, ^10^ For quality assurance, plans were assessed from all centres for protocol compliance and dosimetric consistency.^10^, ^11^

Acute side-effects were graded weekly with National Cancer Institute Common Toxicity Criteria (version 3)^12^ during radiotherapy and until 8 weeks after treatment. Late radiotherapy side-effects were assessed with the Late Effects of Normal Tissues Subjective-Objective Management Analytic (LENT SOMA)^13^, ^14^ and the Radiation Therapy Oncology Group (RTOG)^15^ scoring systems at 3, 6, 12, 18, and 24 months after radiotherapy. Salivary flow measurements were done before radiotherapy, at week 4 of radiotherapy, and at 2 weeks, 3, 6, 12, 18, and 24 months after radiotherapy. Unstimulated and sodium-citrate-stimulated parotid saliva from each parotid duct orifice and floor of mouth saliva were collected by standard methods.^7^, ^8^ After treatment, clinical follow-up was monthly in year 1, every 8 weeks in year 2, then every 3–6 months until the end of year 5. Assessments were not blinded to treatment allocation.

Patient-reported QoL was collected with questionnaire booklets that contained the European Organization for Research and Treatment of Cancer (EORTC) QLQC30 quality-of-life instrument^16^ (which measures generic cancer-related QoL), the associated head and neck specific module HN35,^17^ and the modified xerostomia questionnaire.^8^ Patients completed the baseline booklet in the clinic before randomisation. Follow-up booklets were sent directly to the patients' homes at 2 weeks, 3, 6, 12, 18, and 24 months after radiotherapy.

Our primary objective was to assess late side-effects. Our primary endpoint, agreed in discussion with the independent trial steering committee, was the proportion of patients with xerostomia of grade 2 or worse by the LENT SOMA subjective side-effect scale 1 year after treatment. This endpoint was chosen because it assesses an abnormal symptom (ie, “partial but persistent or complete dryness” or worse) measured by a reliable and sensitive method for scoring late side-effects in HNSCC.^18^ We decided on 12 months a priori as a clinically appropriate time at which to make a valid assessment of late effects.

Secondary endpoints were the proportion of patients with any measurable salivary flow after radiotherapy, acute and other late radiation side-effects, QoL that included xerostomia-related QoL as measured by the modified xerostomia questionnaire, locoregional progression-free survival (PFS), and overall survival. We defined locoregional PFS as time from randomisation to locoregional recurrence or progressive disease as defined by Response Evaluation Criteria in Solid Tumours.^19^ We defined overall survival as time from randomisation to death from any cause.

### Statistical analysis

Phase 2 studies had reported reduction in salivary flow rates of 90% at 1–3 months compared with preradiotherapy rates with conventional therapy and of 40% with IMRT.^7^, ^20^, ^21^, ^22^ If we assume a 1-year xerostomia rate of 90% in the conventional radiotherapy group^23^ a sample size of 84 patients is needed to detect a 30% absolute difference in LENT SOMA of grade 2 or worse xerostomia between the study groups (90% power, 5% two-sided significance). In March, 2007, the independent data monitoring committee and the trial steering committee approved an increase in the target sample size to 84 evaluable patients (ie, alive 1 year after the end of radiotherapy) that was anticipated to be achievable with 100 randomly assigned patients. In December, 2007, both committees approved closure of recruitment after 94 patients had been randomly assigned to the study groups with the expectation that this would provide sufficient evaluable patients to allow robust statistical analysis. Our trial was not powered to reliably assess small differences in locoregional PFS or overall survival, although these are reported for completeness.

Our analysis was done on an intention-to-treat basis, with all patients who had a 12-month xerostomia assessment included. We compared the proportion of patients with grade 2 or worse xerostomia between groups with a χ^2^ test. We assessed the sensitivity of results by repeating analyses of the primary endpoint with patients censored 1 month before any disease recurrence, in case recurrence could adversely affect salivary flow, and by excluding patients whose side-effect assessment was not within 2 months either side of its expected date. We have not presented these sensitivity analyses because they gave similar results to the main analysis. Odds of grade 2 or worse xerostomia at 12 and 24 months were calculated with a logistic-regression model. We present unadjusted odds ratios (ORs) and ORs adjusted for tumour site (oropharynx or hypopharynx), stage of disease (1 and 2 or 3 and 4), and radiotherapy indication (radical or postoperative). All other analyses are unadjusted.

We compared the proportions of patients with any measurable saliva flow and proportions ever reporting grade 2 or worse acute and late side-effects between treatment groups with Fisher's exact tests. For LENT SOMA scales, we used the maximum of the subjective, objective, management, and analytic component scores. We calculated CIs for differences in proportion between groups with a normal approximation. To make some adjustment for multiple testing we used a significance level of 1% for all secondary side-effects, sialometry, and QoL endpoints and accordingly we provide 99% CIs. Acute and late side-effects in our report were those where side-effects of grade 2 or worse were experienced by at least 20% of patients in either group or those where proportions were significantly different between treatment groups.

We calculated QoL scores with standard algorithms with a higher score suggesting poorer QoL on all scales except EORTC global health status, where a higher score suggests better QoL.^24^ We deemed differences in EORTC QoL scores of 10 points or more clinically significant in line with EORTC guidelines.^25^ The primary QoL analysis included all completed questionnaires. We did a sensitivity analysis after censoring at 1 month before disease recurrence or progressive disease. We compared mean changes in EORTC QoL and xerostomia questionnaire item scores from baseline between groups by two-sample *t* tests.

We used generalised estimating equations (GEE), adjusting for the correlations in multiple measurements from the same patient (with an exchangeable correlation matrix) to account for the longitudinal nature of the xerostomia and QoL data. A pragmatic approach to modelling was taken, with treatment-by-time interaction terms included if they were identified in advance as clinically relevant or they were statistically significant. A GEE logistic regression model was fitted with xerostomia (grades 0 and 1 *vs* grades 2–4) as the response and allocated treatment, days since the completion of radiotherapy, and the interaction between the two as covariates. QoL GEE models also included terms for baseline score for the item of interest.

For survival-related endpoints, alive and disease-free patients were censored at date of last follow-up. We compared treatment groups with the log-rank test. Hazard ratios (HRs) with 95% CIs were obtained from Cox proportional hazards regression models with HRs of less than one favouring IMRT. The proportionality assumption of the Cox model held when tested with Schoenfeld residuals.

Our analyses were based on a database snapshot frozen on May 14, 2010, and were done in STATA version 10. ICR-CTSU had overall responsibility for trial coordination. Data collation, central statistical monitoring of data, and all interim and final analyses were performed at ICR-CTSU. The trial management group was responsible for the day to day running of the trial. The trial was overseen by an independent trial steering committee. The independent data monitoring committee regularly reviewed emerging safety and efficacy data in confidence. This study is registered as an International Standard Randomised Controlled Trial, number ISRCTN48243537.

### Role of the funding source

The funding source provided peer-reviewed approval for the trial but had no other role in study design, collection, analysis, interpretation of data, or writing of the report. The corresponding author had full access to all the data in the study and had final responsibility for the decision to submit for publication. JPM, RPA'H, and EH also had full access to all the data.

## Results

**Figure 1:**
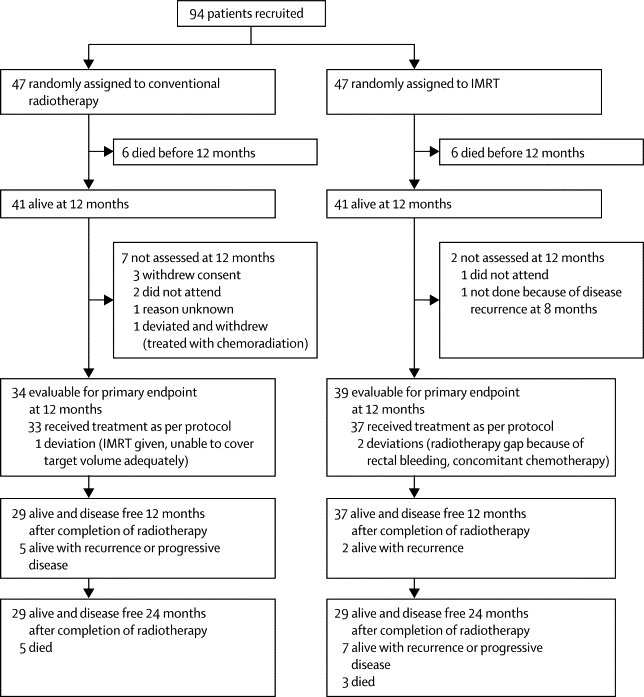
Study profile IMRT=intensity-modulated radiotherapy.

Figure 1 shows the trial profile. We randomly allocated 94 patients from six UK radiotherapy centres to treatment with either IMRT or conventional radiotherapy—47 patients to each group. One patient assigned to the conventional radiotherapy group was deemed ineligible because they were due to be treated with chemoradiation (no follow-up data are available for this patient). Table 1 shows the patient and tumour characteristics at baseline and treatment details. 39 patients (41%) received neoadjuvant chemotherapy (details of specific chemotherapy drugs and doses were not collected). Mean dose to the whole contralateral parotid was significantly less in the IMRT group (p<0·0001; table 1). 45 of 47 patients randomly allocated to receive conventional radiotherapy and 45 of 47 randomly assigned to receive IMRT completed radiotherapy as per protocol; 33 of the 34 patients evaluable for the primary outcome in the conventional radiotherapy group and 37 of 39 patients evaluable for the primary endpoint in the IMRT group completed radiotherapy as per protocol (figure 1). Median follow-up in alive patients was 44·0 months (IQR 30·0–59·7).

**Table 1:** Baseline characteristics and treatment details

		**Conventional radiotherapy (n=47)**	**IMRT (n=47)**
Mean age at randomisation (years)		57·3 (10·2; 37·5–82·8)	59·5 (9·2; 44·1–77·1)
Number of women		12 (26%)	14 (30%)
WHO performance status			
	0	42 (89%)	41 (87%)
	1	5 (11%)	6 (13%)
Tumour site			
	Oropharynx	40 (85%)	40 (85%)
	Hypopharynx	7 (15%)	7 (15%)
Tumour stage			
	T1	6 (13%)	6 (13%)
	T2	27 (57%)	22 (47%)
	T3	11 (23%)	16 (34%)
	T4	3 (6%)	3 (6%)
Nodal stage			
	N0	16 (34%)	23 (49%)
	N1	9 (19%)	15 (32%)
	N2a	7 (15%)	2 (4%)
	N2b	10 (21%)	6 (13%)
	N2c	1 (2%)	0
	N2 (unknown)	1 (2%)	1 (2%)
	N3	3 (6%)	0
AJCC* stage			
	1 and 2	8 (17%)	15 (32%)
	3 and 4	39 (83%)	32 (68%)
Neoadjuvant chemotherapy			
	Yes	19 (40%)	20 (43%)
	No	28 (60%)	27 (57%)
Type of radiotherapy			
	Primary	32 (68%)	39 (83%)
	Postoperative	15 (32%)	8 (17%)
Radiotherapy dose (Gy)			
	Median dose to primary tumour and involved nodes	65·0 (65·0–65·0; 44)	65·0 (65·0–65·0; 47)
	Median dose to elective nodes	50·0 (50·0–50·1; 43)	54·0 (54·0–54·1; 47)
	Mean contralateral parotid dose†	61·0 (54·6–63·8; 43)	25·4 (23·2–28·0; 46)
	Mean ipsilateral parotid dose†	61·0 (57·0–64·4; 43)	47·6 (39·9–54·5; 46)

Data are mean (SD; range), n (%), or median (IQR; n). IMRT=intensity-modulated radiotherapy.

* American Joint Committee on Cancer—groupings based on TNM staging data collected.

† Mann-Whitney test p<0·0001.

**Figure 2:**
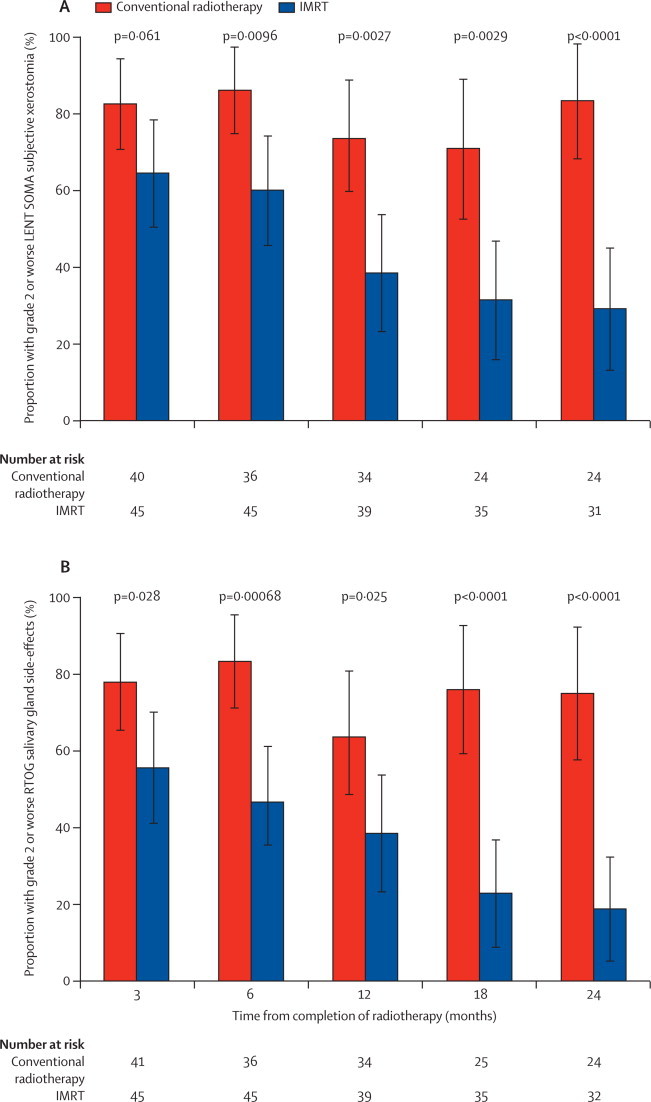
Proportion of patients reporting grade 2 or worse LENT SOMA subjective xerostomia and RTOG salivary gland side-effects p values quoted compare proportions with grade 2 or worse side-effects in each group with a χ^2^ test. Error bars represent 95% CIs. IMRT=intensity-modulated radiotherapy. LENT SOMA=Late Effects of Normal Tissues Subjective-Objective Management Analytic. RTOG=Radiation Therapy Oncology Group.

At each timepoint from 3 to 24 months, a smaller proportion of IMRT patients reported grade 2 or worse LENT SOMA subjective xerostomia compared with conventional radiotherapy (figure 2).

Of the 76 patients who had grade 2 or worse xerostomia during their follow-up, 62 (82%) first reported symptoms at 3 months: 33 (87%) of 38 patients in the conventional radiotherapy group versus 29 (76%) of 38 in the IMRT group. At 12 months, there were significantly fewer cases of xerostomia in the IMRT group (25 [74%, 95% CI 56 to 87] of 34 in the conventional radiotherapy group *vs* 15 [38%, 23 to 55] of 39 in the IMRT group), and the absolute reduction was 35% (95% CI 14 to 56; p=0·0027). At 24 months, 20 (83%, 63 to 95) of 24 patients in the conventional radiotherapy group reported xerostomia versus nine (29%, 14 to 48) of 31 in the IMRT group, and the absolute reduction was 54% (32 to 76; p<0·0001). These differences equate to ORs of 0·23 (0·08 to 0·61) at 12 months and 0·08 (0·02 to 0·31) at 24 months. Adjusted ORs were 0·23 (0·08 to 0·65) at 12 months and 0·05 (0·01 to 0·26) at 24 months. Exploratory GEE analyses showed similar patterns to other analyses presented here (data not shown). The proportion of patients that reported grade 2 or worse xerostomia at 12 months did not differ by tumour site, radiotherapy indication (primary *vs* postoperative), stage of disease, or use of neoadjuvant chemotherapy (data not shown). A similar pattern was seen over time and between treatment groups when xerostomia was scored with the RTOG scale (figure 2).

The only recorded acute adverse event of grade 2 or worse to differ between treatment groups (at the 1% significance level) was fatigue (table 2): 18 (41%; 99% CI 23 to 61) of 44 patients in the conventional radiotherapy group versus 35 (74%; 55 to 89) of 47 in the IMRT group (p=0·0015). Of note, at 12 months, grade 3 or worse dysphagia was reported by two (5%) of 40 patients in the conventional radiotherapy group and four (9%) of 46 in the IMRT group.

**Table 2:** Maximum acute and late side-effect grades by treatment group

	**Conventional radiotherapy**						**IMRT**					
	N	Grade 0	Grade 1	Grade 2	Grade 3	Grade 4	N	Grade 0	Grade 1	Grade 2	Grade 3	Grade 4
Acute side-effects*												
Mucositis/stomatitis (clinical)	44	0	1 (2%)	16 (36%)	27 (61%)	0	46	1 (2%)	2 (4%)	14 (30%)	29 (63%)	0
Rash (dermatitis)†	44	0	3 (7%)	17 (39%)	24 (55%)	0	47	1 (2%)	9 (19%)	21 (45%)	15 (32%)	1 (2%)
Mucositis/stomatitis (functional/symptomatic)	39	1 (3%)	0	21 (54%)	17 (44%)	0	40	2 (5%)	3 (8%)	11 (28%)	24 (60%)	0
Dysphagia	44	0	1 (2%)	26 (59%)	17 (39%)	0	47	1 (2%)	6 (13%)	17 (36%)	23 (49%)	0
Pain	44	0	5 (11%)	23 (52%)	16 (36%)	0	47	1 (2%)	10 (21%)	19 (40%)	16 (34%)	1 (2%)
Fatigue‡	44	0	26 (59%)	12 (27%)	6 (14%)	0	47	2 (4%)	10 (21%)	23 (49%)	12 (26%)	0
Xerostomia	44	0	4 (9%)	35 (80%)	5 (11%)	..	47	0	14 (30%)	20 (43%)	13 (28%)	..
Salivary gland changes	44	0	2 (5%)	40 (91%)	2 (5%)	0	47	0	11 (23%)	30 (64%)	6 (13%)	0
Weight loss	40	2 (5%)	23 (58%)	14 (35%)	1 (3%)	..	44	9 (20%)	14 (32%)	19 (43%)	2 (5%)	..
Hair loss/alopecia	44	14 (32%)	22 (50%)	8 (18%)	0	..	47	7 (15%)	27 (57%)	12 (26%)	1 (2%)	..
**RTOG late side-effects**§												
Salivary gland¶	42	1 (2%)	3 (7%)	26 (62%)	12 (29%)	0	46	0	12 (26%)	32 (70%)	2 (4%)	0
Mucous membranes	42	1 (2%)	23 (55%)	17 (40%)	1 (2%)	0	46	4 (9%)	29 (63%)	12 (26%)	1 (2%)	0
Oesophagus	42	22 (52%)	11 (26%)	8 (19%)	1 (2%)	0	46	19 (41%)	17 (37%)	8 (17%)	2 (4%)	0
Joint (temporomandibular joint disorder)	42	22 (52%)	11 (26%)	9 (21%)	0	0	46	31 (67%)	11 (24%)	3 (7%)	1 (2%)	0
**LENT SOMA late side-effects**§												
Salivary gland‡,‖(xerostomia‡)	41	0	3 (7%)	12 (29%)	14 (34%)	12 (29%)	46	0	8 (17%)	19 (41%)	15 (33%)	4 (9%)
		0	3 (7%)	19 (46%)	14 (34%)	5 (12%)		0	8 (17%)	31 (67%)	4 (9%)	3 (7%)
Mucosa**	41	1 (2%)	9 (22%)	17 (41%)	9 (22%)	5 (12%)	46	1 (2%)	19 (41%)	11 (24%)	11 (24%)	4 (9%)
Oesophagus†† (dysphagia)	41	15 (37%)	15 (37%)	4 (10%)	5 (12%)	2 (5%)	46	20 (43%)	16 (35%)	4 (9%)	4 (9%)	2 (4%)
		20 (49%)	16 (39%)	3 (7%)	2 (5%)	0		21 (46%)	16 (35%)	5 (11%)	3 (7%)	1 (2%)
Skin‡‡	41	5 (12%)	19 (46%)	11 (27%)	5 (12%)	1 (2%)	46	10 (22%)	24 (52%)	10 (22%)	2 (4%)	0
Larynx§§	41	16 (39%)	15 (37%)	7 (17%)	2 (5%)	1 (2%)	46	16 (35%)	22 (48%)	8 (17%)	0	0
Mandible¶¶	41	13 (32%)	16 (39%)	9 (22%)	3 (7%)	0	46	19 (41%)	11 (24%)	12 (26%)	3 (7%)	1 (2%)
Ear‖‖	41	19 (46%)	12 (29%)	7 (17%)	3 (7%)	0	46	27 (59%)	13 (28%)	6 (13%)	0	0

Data are n (%). IMRT=intensity-modulated radiotherapy. RTOG=Radiation Therapy Oncology Group. LENT SOMA=Late Effects of Normal Tissues Subjective-Objective Management Analytic.

* Maximum Common Toxicity Criteria score during and up to 8 weeks post radiotherapy.

† p for trend 0·01<p≤0·05.

‡ p for trend 0·001<p≤0·01.

§ Maximum score between 3 and 24 months post radiotherapy.

¶ p for trend p≤0·001.

‖ Worst of subjective (xerostomia), objective (saliva flow), and management (xerostomia) grades.

** Worst of subjective (pain, dysphagia, taste alteration), objective (mucosal integrity, weight), and management (pain, ulcer, dysphagia, taste alteration) grades.

†† Worst of subjective (dysphagia, pain), objective (weight loss, stricture, ulceration, bleeding, anaemia), and management (dysphagia/stricture, weight loss, pain/ulceration, bleeding) grades.

‡‡ Worst of subjective (roughness, sensation), objective (oedema, alopecia, pigmentation change, ulcer/necrosis, telangiectasia, fibrosis/scar, atrophy/contraction), and management (dryness, sensation, ulcer, oedema, fibrosis/scar) grades.

§§ Worst of subjective (pain, voice hoarseness, breathing), objective (oedema, mucosal integrity, respiration), and management (pain, hoarseness, respiration) grades.

¶¶ Worst of subjective (pain, mastication, denture use, trismus), objective (exposed bone, trismus), and management (pain, bone, trismus/mastication) grades.

‖‖ Worst of subjective (pain, tinnitus, hearing), objective (skin, hearing), and management (pain, skin, hearing loss) grades.

We recorded baseline sialometry in 80 patients, all of whom had measurable salivary flow. At 12 months unstimulated saliva flow from the contralateral parotid gland was noted in 16 (47%) of 34 patients in the IMRT group compared with none of 25 in the conventional radiotherapy group (p<0·0001). Corresponding data at 24 months were seven (44%) of 16 in the IMRT group versus none of 15 in the conventional radiotherapy group (p=0·0068). Significant differences were also noted in stimulated saliva flow from the contralateral parotid at 12 months (p<0·0001). No significant differences between the random assigned groups were seen in proportions with unstimulated or stimulated flow from either the ipsilateral parotid or floor of mouth. Strong concordance was noted between measurable contralateral saliva flow and grade 2 or worse xerostomia (table 3).

**Table 3:** Concordance between unstimulated contralateral saliva flow and LENT SOMA subjective xerostomia at 12 months

	**Conventional radiotherapy**		**IMRT**	
	No measurable salivary flow* (n=25)	Measurable salivary flow (n=0)	No measurable salivary flow (n=18)	Measurable salivary flow (n=16)
Subjective xerostomia better than grade 2	6 (24%)	0	10 (56%)	12 (75%)
Subjective xerostomia grade 2 or worse	19 (76%)	0	8 (44%)	4 (25%)

Fisher's exact test for association (treatment groups combined) p=0·018. LENT SOMA=Late Effects of Normal Tissues Subjective-Objective Management Analytic. IMRT=intensity-modulated radiotherapy.

* Measurable salivary flow was defined as any saliva collected from the Lashley cup apparatus.

Mean changes in global health status from baseline to 12 months were 1·1 (99% CI −9·9 to 12·1) for conventional radiotherapy versus 3·0 (–11·9 to 17·9; p=0·78) for IMRT. Changes at 24 months were −2·8 (–17·1 to 11·6) for conventional radiotherapy versus 8·3 (–6·6 to 23·2) for IMRT, corresponding to a between group difference in change scores of 11·1 (–9·0 to 31·2; p=0·14). No statistically significant differences in change from baseline between groups were noted for any QLQC30 subscale scores (data not shown).

**Figure 3:**
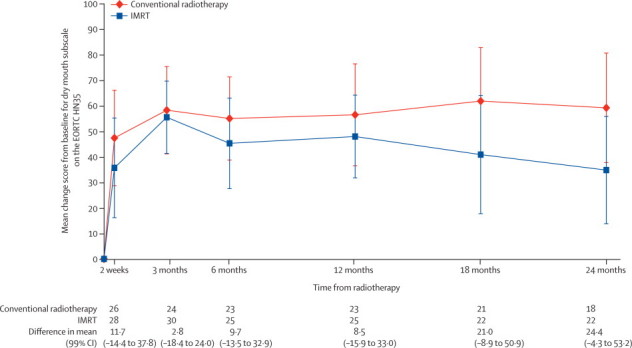
Mean EORTC HN35 dry mouth subscale score changes from baseline IMRT=intensity-modulated radiotherapy. EORTC HN35=European Organization for Research and Treatment of Cancer head and neck specific module HN35.

In both study groups, HN35 subscale scores for dry mouth, senses, and sticky saliva were significantly worse than baseline at 12 months. Figure 3 shows mean increases from baseline from 2 weeks to 24 months in dry mouth subscale score, by treatment group. Mean increases from baseline at 12 months in the dry mouth subscale were 56·5 (99% CI 36·5 to 76·5; p<0·0001) for conventional radiotherapy and 48·0 (31·8 to 64·2; p<0·0001) for IMRT. Mean increases at 24 months were 59·3 (37·8 to 80·7; p<0·0001) for conventional radiotherapy and 34·8 (13·8 to 55·9; p<0·0001) for IMRT. At both time points, smaller score changes were noted in the IMRT group than in the conventional radiotherapy group, although these were not significant at the 1% level.

In the GEE model for dry mouth the main treatment coefficient was −6·6 (99% CI −21·5 to 8·3; p=0·25) with a treatment-by-time interaction term of −0·03 (–0·06 to 0·00; p=0·017), suggesting the difference in dry mouth between treatment groups increases over time. Censoring at recurrence had a negligible effect on QoL results, although the interaction term from the GEE analysis became less statistically significant (coefficient −0·02; p=0·080).

The xerostomia questionnaire was only completed by 39 patients at baseline and 12 months and by 33 patients at baseline and 24 months (compared with 73 reporting the primary endpoint at 12 months and 55 at 24 months). In both treatment groups all eight xerostomia questionnaire items were significantly worse at 12 and 24 months than at baseline and although the changes were smaller in the IMRT group, no statistically significant differences between group changes were noted (webappendix p 1).

**Figure 4:**
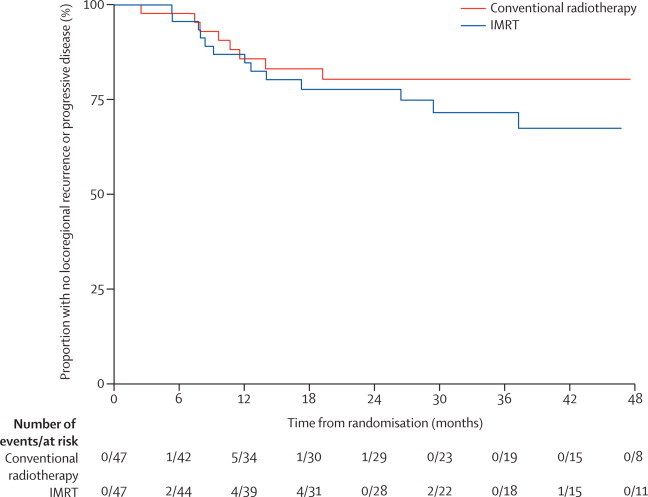
Kaplan-Meier plot of locoregional progression-free survival by treatment group Hazard ratio 1·53 (95% CI 0·63 to 3·70). Log-rank test p=0·34. 2-year locoregional progression-free survival estimates for conventional radiotherapy 80% (95% CI 65 to 90) and for IMRT 78% (62 to 87); absolute difference 3% (–15 to 20). IMRT=intensity-modulated radiotherapy.

Overall, there were seven locoregional recurrences in the conventional radiotherapy group: five in the high-dose volume and two in both the high-dose volume and electively irradiated neck. In the IMRT group there were 12 locoregional recurrences: 11 in the high-dose volume and one in the electively irradiated neck. No patients had a recurrence in the spared parotid tissue. 2-year locoregional PFS was 80% (95% CI 65 to 90) in the conventional radiotherapy group and 78% (62 to 87) in the IMRT group (absolute difference 3%, 95% CI −15 to 20; HR 1·53, 95% CI 0·63 to 3·70; log-rank p=0·34; figure 4).

32 deaths have been reported so far (18 in the conventional radiotherapy group *vs* 14 in the IMRT group; HR for overall survival 0·68, 95% CI 0·34 to 1·37). Of these deaths, 20 were due to head and neck cancer (ten in the conventional radiotherapy group *vs* ten in the IMRT group). Other causes of death in the conventional radiotherapy group were second (non-head-and-neck) primary cancer (four patients), cardiac (two), gastrointestinal complications (one), and suicide (one); and in the IMRT group were infection (two), second primary cancer (one), and gastrointestinal complications (one). Estimated 2-year overall survival was 76% (95% CI 60 to 86) with conventional radiotherapy and 78% (63 to 88) with IMRT (absolute difference 2%, 95% CI −20 to 16).

## Discussion

Our trial showed a significant reduction of radiation-induced xerostomia for patients treated with IMRT compared with conventional radiotherapy by use of both LENT SOMA and RTOG scales. Furthermore, we showed recovery of saliva flow by quantitative measurements, and improvements on QoL measures associated with xerostomia. To our knowledge our trial is the first to show that parotid-sparing IMRT reduces xerostomia in HNSCC (panel). A consistently higher QLQC30 Global and HN35 dry mouth QoL score was reported in patients who received IMRT; between group differences at 24 months were clinically but not statistically significant. Xerostomia questionnaire results showed changes in favour of IMRT in all eight questions but these differences were not large enough to reach statistical significance, probably because of the small number of patients that completed this questionnaire. Although an association between measurable saliva flow and presence of grade 2 or worse xerostomia was recorded, there was not perfect concordance. We postulate that this could be because of differences in patient perception of the xerostomia symptom or because of other factors such as submandibular gland or oral cavity dose or comorbidity. Detailed analyses of the distribution of dose to the salivary tissue including parotid glands and other minor salivary glands, and its correlation with clinical outcomes are ongoing. Initial results suggest that there is no correlation between submandibular gland dose and xerostomia.

Panel **Research in context**

**Systematic review**

Intensity modulated radiotherapy (IMRT) allows focused radiation delivery to tumours. In patients with head and neck cancer it has been used to reduce the irradiation of salivary tissue to prevent radiation-induced xerostomia. Before the design of our randomised trial, a few small single centre experiences had been published and a review of the published work on IMRT had been done.^26^ No randomised trials were identified. During the recruitment period of the PARSPORT trial two smaller randomised trials were reported in nasopharyngeal cancer from centres in Asia, and several other single institutional experiences were reported.^27^, ^28^

**Interpretation**

Our trial is the largest randomised trial of IMRT in head and neck cancer, and the only trial addressing squamous-cell carcinoma, the predominant form seen worldwide. Our trial shows that IMRT reduces patient-reported xerostomia, allows recovery of salivary flow, and improves quality of life after treatment compared with conventional radiotherapy.

A limitation of our trial was that it was not possible to mask the treatments from patients or clinicians because of differences in treatment delivery. However, results that relate to multiple secondary endpoints support the primary analysis and the size of the observed effect is unlikely to be due entirely to assessment or reporting bias. After our trial was designed, several small non-randomised studies^29^, ^30^, ^31^, ^32^, ^33^, ^34^, ^35^ and one case-control study^36^ of parotid-sparing IMRT have been published with a range of endpoints including saliva flow rate, patient-reported symptoms, and QoL. These studies reported apparent improvements for IMRT over conventional radiotherapy. Two small single-institution randomised phase 3 trials of IMRT in nasopharyngeal cancer have also reported benefits of IMRT over conventional radiotherapy. Pow and colleagues^37^ reported an increase in stimulated whole saliva flow rate in patients receiving IMRT in a randomised trial of 51 patients with early-stage nasopharynx cancer. QoL was assessed with EORTC QLQC30, HN35, and the SF36 health survey and although QoL scores for some domains were better for IMRT patients, no improvements in patient-reported dry mouth symptoms on the HN35 questionnaire were noted. Kam and colleagues^38^ reported a reduction in observer-rated severe xerostomia (RTOG grade 2 or worse) with IMRT (39% *vs* 82%; p=0·001) in 60 patients with early-stage nasopharyngeal cancer. The results of the PARSPORT trial are thus likely to be generalisable to all head and neck tumours for which conventional radiotherapy is used.

In our study, fewer cases of acute dermatitis were recorded in patients treated with IMRT than in those treated with conventional radiotherapy, although differences were not statistically significant at the 1% level, probably because of reduced dose to skin. The proportions of patients that reported grade 2 or worse acute xerostomia and grade 2 or worse salivary gland changes also showed reductions, albeit not statistically significant (table 2). Late xerostomia side-effects thus accord with acute side-effects; this suggests that late radiation-induced xerostomia is a consequential effect. We did not attempt to spare the submandibular or mucosal minor salivary glands within the planning target volume in our trial. It is possible that further reductions in severe xerostomia can be achieved by sparing these tissues, but this might risk underdosing crucial target tissues. Unexpectedly, acute fatigue was greater in patients treated with IMRT, which could be due to the greater radiation dose to non-tumour tissues. In an unplanned dosimetry review in a subset of patients, mean radiation doses to the posterior fossa were 20–30 Gy in the patients treated with IMRT compared with about 6 Gy in patients treated with conventional radiotherapy, which could account for the recorded difference in acute radiation fatigue. Late fatigue data were not collected because lethargy is not a recognised long-term side-effect of radiotherapy. There was no significant association between the giving of neoadjuvant chemotherapy and either acute fatigue or xerostomia (data not shown). The addition of concurrent chemotherapy to altered fractionation radiotherapy remains experimental and was not used in our study. Further research is needed to establish the effect of concurrent chemotherapy on xerostomia. Apart from salivary gland changes and radiation-induced xerostomia, other late side-effects of conventional radiotherapy were not altered by IMRT.

Our trial was too small to detect small differences in, or conclude non-inferiority of, locoregional PFS or overall survival. Although patients continue to be followed up for long-term survival, to show non-inferiority in overall survival to no more than 5% at 2 years (80% power, one-sided 5% significance) would need a randomised controlled trial of more than 900 patients. In this, and other, head and neck IMRT studies most tumour recurrences happen within the high-dose volume. Recurrences have not been noted in the spared parotid tissue in patients treated with IMRT or surgery,^21^, ^39^ suggesting that a large study to show non-inferiority in this tumour type is probably both impractical and inappropriate. Our trial has shown a clinically and statistically significant reduction in xerostomia, improved salivary flow, and improved QoL, and thus strongly supports a role for IMRT in HNSCC.

## Web Extra Material

Supplementary webappendix
