# Supplementary material for: Parotid-sparing intensity modulated versus conventional radiotherapy in head and neck cancer (PARSPORT): a phase 3 multicentre randomised controlled trial
Source: Lancet Oncol. 2011 Feb;12(2):127–36. doi: 10.1016/S1470-2045(10)70290-4 (PMC3033533; doi:10.1016/S1470-2045(10)70290-4)
Supplement: Supplementary webappendix [file mmc1.pdf]

THE LANCET Oncology

Supplementary webappendix

This webappendix formed part of the original submission and has been peer reviewed. We post it as supplied by the authors.

Supplement to: Nutting CM, Morden JP, Harrington KJ, et al. Parotid-sparing intensity modulated versus conventional radiotherapy in head and neck cancer (PARSPORT): a phase 3 multicentre randomised controlled trial. *Lancet Oncol* 2011; published online Jan 13. DOI:10.1016/S1470-2045(10)70290-4.

Web Appendix – "Results of a Phase III Multi-Center Randomized Controlled Trial of Parotid-Sparing Intensity Modulated versus Conventional Radiotherapy in Head and Neck Cancer"

Table A: Mean changes from baseline, and differences between treatment groups in Modified Xerostomia questionnaire items

XQ item	CRT				IMRT				Difference between arms (CRT - IMRT)	99% CI	
	n	Mean deterioration from baseline	99% CI Lower	99% CI Upper	n	Mean deterioration from baseline	99% CI Lower	99% CI Upper		Lower	Upper
**Baseline to 12 months**											
1) Difficulty speaking	17	3.6	1.8	5.5	22	2	0.1	3.9	1.6	-0.9	4.2
2) Difficulty chewing	17	5.6	3.5	7.7	21	4.1	2	6.2	1.5	-1.4	4.4
3) Difficulty swallowing	17	5.3	3.1	7.5	21	4.2	2	6.5	1.1	-2	4.1
4) Dryness when eating	17	5.3	3.2	7.4	21	4.1	2	6.2	1.2	-1.7	4
5) Dryness when not eating	17	4.3	2.3	6.3	22	3.2	1.4	5	1.1	-1.4	3.7
6) Sipping liquids to aid swallowing	17	5.4	2.9	7.9	21	4.4	1.7	7.1	1	-2.5	4.6
7) Sipping liquids when not eating	17	4	1.4	6.6	22	2.9	0.9	4.9	1.1	-2	4.1
8) Sleeping problems due to dryness	17	3.2	0.5	5.8	22	2.2	0.2	4.2	1	-2.1	4.1
**Baseline to 24 months**											
1) Difficulty speaking	13	3.9	1.6	6.3	20	1.9	-0.4	4.1	2.1	-1.1	5.2
2) Difficulty chewing	13	5.7	2.9	8.5	20	3.7	1	6.3	2	-1.7	5.8
3) Difficulty swallowing	13	5.5	2.7	8.4	20	4.2	1.7	6.7	1.3	-2.3	4.9
4) Dryness when eating	13	5.3	2.4	8.2	20	4.1	1.7	6.4	1.3	-2.3	4.8
5) Dryness when not eating	13	4.2	1.3	7	20	3	0.9	5	1.2	-2	4.5
6) Sipping liquids to aid swallowing	12	6	3.2	8.8	20	4	1.2	6.8	2	-1.9	5.9
7) Sipping liquids when not eating	13	4.1	1.3	6.9	20	1.9	0	3.7	2.2	-0.7	5.2
8) Sleeping problems due to dryness	13	2.1	-0.8	5	20	1.4	-0.8	3.6	0.7	-2.7	4.1

PARSPORT Trialists

Participating Centres: We thank the patients and all the investigators and research support staff at the participating centers.

Principal and main co-investigators according to center (number of patients recruited) were: Royal Marsden Hospital NHS Foundation Trust, Sutton (28) and London (45) – C Nutting, K Harrington, K Newbold, S Bhide, T Guerrero Urbano; Ipswich Hospital (6) – C Scrase; Christie Hospital Manchester (7) – B Yap, N Slevin, A Sykes; Addenbrookes Hospital NHS Foundation Trust (2) – S Jefferies, R Benson; University Hospital of North Staffordshire (6) – F Adab.

Trial Management Group: In addition to the authors the following are members of the Trial Management Group; D Carnell (University College Hospital, London), C Coyle (St James University Hospital, Leeds), C Mackenzie (Ipswich Hospital), G Robertson, M Robinson (Weston Park Hospital, Sheffield), A Sibtain (St Bartholomews Hospital, London), N Slevin (Christie Hospital, Manchester), J Swinscoe (Weston Park Hospital, Sheffield), A Sykes (Christie Hospital, Manchester) and G Webster (Christie Hospital, Manchester).

Trial Steering Committee: S Bentzen (Chair, University Of Wisconsin Medical School, Madison, USA), S Rogers (University of Liverpool), V Gregoire (,Belgium) and M Henk (Royal Marden Hospital NHS Foundation Trust, Sutton, retired).

Independent Data Monitoring Committee: P Hoskin (Chair, Mount Vernon Hospital, Middlesex), M Saunders (University College Hospital London, retired) and W Qian (MRC Clinical Trials Unit, London)

ICR-CTSU: In addition to the authors the following were also members of ICR-CTSU and contributed to co-ordination and analysis of the trial, S Ghuman, L Lloyd, S Patel, C Peckitt and MS Rogers.
